# Protective effect of intraoperative nerve monitoring against recurrent laryngeal nerve injury during re-exploration of the thyroid

**DOI:** 10.1186/1477-7819-11-94

**Published:** 2013-04-23

**Authors:** Yu-Chuan Chuang, Shih-Ming Huang

**Affiliations:** 1 Department of Surgery, National Cheng Kung University Hospital, Tainan, 704, Taiwan

**Keywords:** Intraoperative neuromonitoring, Re-operation, Re-exploration, Recurrent laryngeal nerve injury

## Abstract

**Background:**

Previous thyroid or parathyroid surgery induces scarring or distorts anatomy, and increases the risk of recurrent laryngeal nerve (RLN) injury for a reoperation. The benefit of intraoperative nerve monitoring (IONM) for re-exploration (a second nerve exploration) and reoperation has not been established.

**Methods:**

Two hundred and ten patients were given a thyroid or parathyroid reoperation at our hospital between 2001 and 2010. Using IONM, we re-explored 56 patients who had been operated on before June 2007. The injury rate in these patients was compared with that of the 15 patients re-explored without IONM between 2001 and 2006.

**Results:**

Of the 70 nerves that were re-explored using IONM, only one was incidentally injured, significantly fewer than the three injured in the 15 nerves re-explored without using IONM (1.43% *vs.* 20%, *P* = 0.0164).

**Conclusions:**

IONM helped prevent RLN damage when re-exploring nerves during thyroid and parathyroid surgery. We recommend the routine use of IONM in thyroid and parathyroid reoperations.

## Background

Recurrent laryngeal nerve (RLN) injury is a rare but severe complication of thyroid surgery. A surgeon’s experience and knowledge about anatomy is the keystone of preventing RLN injury [1]. Identifying the RLN visually during surgery can lower the incidence of RLN injury and the permanent injury rate to 0.9% [2-4].

Thyroid surgery induces adjacent tissue scarring and distortion of the anatomy. The distorted anatomy increases the incidence of the RLN injury during subsequent thyroid surgery, even if done by a high-volume surgeon (2% to 12%) [5-7].

Intraoperative nerve monitoring (IONM) is a technique that can help identify or map the course of the RLN nerve and recognize its function [1]. One study [8] reported that in the general population, using IONM provided no significant benefit for preventing permanent RLN injury. However, in some high-risk patients, such as in a reoperation or surgery for malignancy, using IONM might be beneficial, although this is still controversial [1,9-11].

In previous studies [9-11], patients with a reoperation were included, and surgeons counted the total number of nerves during the exploration in the second operation. However, none of these studies definitely pointed out the actual RLN injury rate during the re-exploration. These studies may in their reoperation denominator an RLN that had never been explored before, which would underestimate the actual injury rate during every re-exploration. Also, none of these studies clearly compared the RLN injury rate during re-exploration between groups in which IONM was and was not used. In addition, data on the incidence of RLN injuries in re-explorations done by a single high-volume surgeon before and after the introduction of IONM are lacking.

In this study, we focus on the effect of IONM in the re-exploration of the RLN and the experience of a single high-volume surgeon.

## Methods

### Definitions

‘Reoperation’ means a second or subsequent surgery to correct the same condition or complications of a previous surgery. ‘Re-exploration’ means a second or subsequent nerve exploration for a reoperation.

## Material

An IONM device was introduced to our institute in March 2006. All patients given thyroid reoperations with IONM were included in this study. All the medical records were reviewed retrospectively for gender, age, diagnosis, indications of the first and second surgeries, operative methods of the first and second surgeries, time interval between the two operations, and the signs and symptoms of RLN paresis before and after reoperations. A preoperative laryngoscopy exam was routinely done by an otolaryngologist for every patient given a reoperation.

All reoperations were done by a single high-volume surgeon who had already done more than 4000 thyroid surgeries before we conducted this study. All patients given a reoperation between 2001 and 2006 by the same surgeon without IONM were also reviewed.

Exclusion criteria included unknown methods for the previous surgery and patients who had not had an RLN re-exploration during the reoperation. Patients who had vocal cord palsy from a previous operation, detected in a laryngoscope examination, were also excluded.

Between 2001 and 2010, 210 patients were reoperated on by the surgeon. Only 56 patients given an RLN re-exploration using IONM and 15 patients without IONM were included in this study.

### IONM

The IONM device was set up in a standard manner with two electrodes on the endotracheal tube (NIM EMG Endotracheal Tube; Medtronic, Minneapolis, MN, USA). A four-step procedure was used to ensure that the device was functioning adequately (Table 1) [12,13].

**Table 1:** Four-step intraoperative neuromonitoring (IONM) procedure during thyroid surgery

**Steps**	**Timing**
1	Test vagus nerve before identifying the RLN
2	Test RLN when it is first identified
3	Test RLN^a^ after it is completely dissected from Berry’s ligament
4	Test vagus nerve after complete hemostasis

^a^Test the most proximally exposed point.

RLN, Recurrent laryngeal nerve.

All patients received transverse cervical incision during reoperation. Lateral approach or medial approach is determined by the result of preoperative sonography performed by the surgeon. We routinely checked the vagus nerve, mapped the course of the RLN, and always did IONM stimulation tests before we ligated or transected any tissue during surgery. The stimulation current is set at 1 mA. However, 2 mA is used sometimes to map the route of RLN while not seen very clearly. The normal range of amplitude is 700 to 1500 μv.

### Statistical method

We used Fisher’s exact test for comparing the two-tailed *P* value between the two groups. Significance was set at *P* <0.05.

## Results

Fifty-six patients with a definite re-exploration of the RLN were analyzed. IONM was used to re-explore 70 RLNs. The indications and operative methods of the first and second operations are shown in Table 2.

**Table 2:** Re-exploration of the recurrent laryngeal nerve (RLN) with and without intraoperative neuromonitoring (IONM)

	**With IONM**	**Without IONM**
Total patients (*n*)	56	15
Previous cervical exploration (*n*)	1.25	1.4
Total nerve re-exploration (*n*)	70	15
Male:Female ratio	8:48	4:11
Age (years)	22.8-80.5	29.7-85.0
*Pathology*		
Papillary cancer	23	12
Medullary cancer	1	
Follicular cancer		2
Nodular goiter	20	1
Graves’ disease	12	
Hyperparathyroidism	1	
*Indication of reoperation*		
Malignancy		
Thyroid bed recurrence	8	5
Lymph node recurrence	10	7
Completion thyroidectomy	11	2
Benign		
Recurrent Graves’ disease	4	
Airway compression	7	1
Suspect malignant	16	
Recurrent hyperparathyroidism	1	
*Procedure*		
Total thyroidectomy + LN dissection = > Excision:	9	9
Total thyroidectomy = > LN dissection:	2	3
Total thyroidectomy = > LN dissection + excision	1	
Total thyroidectomy + LN dissection = > Laryngectomy	1	
Total + subtotal thyroidectomy = > Completion thyroidectomy:	17	2
Total + subtotal thyroidectomy = > LN dissection	1	
Total thyroidectomy + LN dissection = > LN dissection	1	
Subtotal thyroidectomy + Trachea resection = > Completion thyroidectomy	1	
Subtotal thyroidectomy = > Lobectomy:	3	1
Bilateral subtotal thyroidectomy = > Lobectomy	10	
Bilateral subtotal thyroidectomy = > total thyroidectomy	2	
Bilateral subtotal thyroidectomy = > total + subtotal thyroidectomy	1	
Bilateral subtotal thyroidectomy = > Excision	1	
Parathyroidectomy = > Excision	1	0

LN, Lymph node.

Of the 70 RLNs re-explored with IONM, three were transected (Table 3): two nerves were intentionally transected and one (1.43%) was accidentally injured. In comparison, of the 15 RLNs re-explored without IONM, three (20%, *P* = 0.0164) nerves were accidentally injured (Tables 2 and 4). The overall injury rate of RLNs during reoperation for this surgeon was 1.90% (4 RLNs in 210 cases).

**Table 3:** **Information on permanent injury of the recurrent laryngeal nerve (RLN) using intraoperative neuromonitoring (IONM) (*n *= 3; 5.36% per patient; 4.29% per nerve and 1.43% of incidental injury, *P *= 0.0164)**

**Age(years)**	**Gender**	**Pathologic diagnosis**	**Previous procedure**	**Indication of reoperation**	**Current procedure**	**Cause of injury**
60.8	Female	PC	To + LNd	T bed	Laryngectomy	Intentional, involved
61.0	Female	PC	To	T bed	Excision	Accidental
38.3	Female	PC	To + LNd	LN rec	Excision	Intentional, involved

LN rec, Lymph node recurrence; LNd, Lymph node dissection; PC, Papillary cancer; T bed, Thyroid bed recurrence; To, Total thyroidectomy.

**Table 4:** **Information on permanent injury of the recurrent laryngeal nerve (RLN) without intraoperative neuromonitoring (IONM) (*n *= 3, 20% per patient and nerve)**

**Age (years)**	**Gender**	**Pathologic diagnosis**	**Previous procedure**	**Indication of reoperation**	**Current procedure**	**Cause of injury**
55.1	Female	PC	To + LNd	T bed	Excision	Accidental
38.3	Female	PC	To + LNd	LN rec	Excision	Accidental
45.0	Female	NG	ST	Airway	Lobectomy	Accidental

Airway, Airway compression; LN rec, Lymph node recurrence; LNd, Lymph node dissection; NG, Nodular goiter; PC, Papillary cancer; ST, Subtotal thyroidectomy; T bed, Thyroid bed recurrence; To, Total thyroidectomy.

We routinely checked the vagus nerve, mapped the course of the RLN, and always did IONM stimulation tests before we ligated or transected any tissue during surgery. However, the IONM device malfunctioned once during an operation, which resulted in an accidental RLN transection. We were unaware of the RLN injury until we used the IONM to check the vagus nerve and received no response. In contrast, the contralateral vagus nerve responded well to stimulation. We identified the transected end of the RLN using a nerve stimulator and did a re-anastomosis with the ansa-cervicalis nerve.

In another patient, one RLN was transected because of a tumor encasement. The short residual nerve end beneath Berry’s ligament was confirmed using a nerve stimulation test and was re-anastomosed with the ansa-cervicalis nerve. The patient had an acceptable voice during follow-up despite laryngoscope-revealed vocal cord palsy.

In a third patient, the RLN was intentionally transected because of recurrent thyroid cancer with tracheal involvement. We initially tried to do a segmental tracheal resection and primary anastomosis to preserve her larynx. However, the RLN lost function during surgery even though the nerve appeared to be visually intact. The injury might have been caused by excessive traction during dissection, but we could not tell whether it was a temporary or permanent injury. For safety, we changed the operation to a total laryngectomy and sacrificed the RLN as well.

## Discussion

In reoperative thyroid surgery, there is higher rate of complications because adjacent tissue is scarred and anatomy is distorted. The scarring makes dissection more difficult, and bleeding further compromises the operation field. Distorted anatomy increases the injury rate of important adjacent structures [1]. Even for an experienced surgeon, reoperative surgery is stressful.

RLN injury in reoperative surgery occurs primarily because of difficulty in identifying the RLN. Identifying the RLN lowers the incidence of permanent RLN injury from 5.2% to 1.2% [4]. In addition, the RLN injury rate in reoperative surgery is around 1% to 12% in different studies [5-7]. IONM helps to identify and map the route of the RLN during reoperation, but whether it prevents RLN injury is still controversial [1,9-11]. One study [10] showed that the rate of RLN injury was higher in a group of reoperative thyroidectomies done without IONM (19%) than in a group done with IONM (7.8%). Another study [1] reported 1.9% and 1.7% permanent RLN injury rates for reoperative thyroidectomies with and without IONM, respectively [1]. However, no study presents the actual RLN injury rate during reoperative thyroid surgery with RLN re-exploration, nor does any show that IONM is beneficial for re-exploring the RLN.

Another study [14] includes 89 and 157 patients with and without IONM usage, respectively. And the incidence of transient nerve palsy rate is higher in the group with IONM usage (6.2 *vs.* 2.5%), but showed no statistically significance. The incidence of permanent injury rate is 0% in the group with IONM usage, and 0.6% in those without. In this study, <10% of the patient was diagnosed as thyroid malignancy and the operation is performed by eight surgeons with different experience. Besides, the patient group is selected according to the surgeon’s preference, which would cause huge selection bias. In our study, patients with IONM usage showed significant benefit in preventing RLN injury and the reason may be that we included more thyroid malignancy in our patient group. In our study, 42.9% of patients with IONM and 80% of patients without IONM are diagnosed with thyroid malignancy and this may increase the difficulty of reoperation due to tumor invasion. All the operations in our study are performed by a single high-volume surgeon and all patients received IONM usage after introduction of IONM, which would significantly reduce bias from patient selection and surgeon’s technique.

In our study, both groups of patients had a definite history of exploration and re-exploration of RLNs. The actual injury rate of each re-exploration was compared. For the group without IONM, the accidental injury rate was 20% (3 of 15 nerves), and for the group with IONM, it was a significantly (*P* <0.05) different 1.43% (1 of 70 nerves). The overall reoperation injury rate in our institute is 1.90% (4 of 210 cases). Although the benefit of using IONM for reoperative thyroid surgery may not be indisputable, we hypothesize that if re-exploration near RLNs is planned and done using IONM, significantly fewer accidental RLN injuries will occur. IONM will also give surgeons more confidence when re-exploring RLNs, as shown with the patients in this study.

Several potential pitfalls of IONM have been reported [15,16], such as device malfunction, improper device setup, misuse of muscle relaxant, anatomic variation of the RLN, and shunt stimulus, which can cause misleading information. Device set-up problems are the most common cause of false IONM results. In the one case of accidental injury in this study, an RLN was transected because the monitor temporarily stopped functioning during surgery. To prevent this from occurring, a standard manual for device set-up and proper endotracheal tube insertion is necessary [13]. However, the possibility that the IONM device will malfunction or require readjustment during the operation still exists. Intermittently stimulating the vagus nerve using a recently designed anchor electrode [17,18] in addition to a conventional handheld bipolar stimulation electrode during surgery may alert the surgeon to device malfunction. In our first case of RLN injury, device malfunction was identified as the main reason of injury. Continuous IONM would gain a lot of benefit in this situation and prevent nerve injury [19].

There are other advantages of using IONM during thyroid surgery. For accidental transection or deliberate sacrifice of the RLN, IONM helps when searching for the transected end. Once the transected end is identified, better re-anastomosis of the RLN can be achieved. In addition, IONM helps identify the transected end of RLNs with very short ends and RLNs that are buried in the Berry’s ligament. This should allow a more precise nerve repair or even re-anastomosis with the ansa cervicalis. However, the actual benefits require more investigation [20]. IONM revealed advantage in endoscopic surgery in animal study, but the benefit in human need further investigation [21].

For patients with bilateral disease or preoperative unilateral RLN paresis, IONM may guide the strategy of the thyroid operation. IONM can be used to monitor the RLN function because that function may still be impaired even though the RLN visually appears to be intact. In one study [22], 33 patients developed unilateral cord paralysis, but only five nerve injuries were recognized during surgery. In another [3], 40 patients developed cord paralysis with only three recognizable injuries. The possible mechanisms include forceps clamping, nerve stretching, electrothermal injury, ligature entrapment, and ischemia. A stretching injury may be the main reason of temporary paresis [12]. If a temporary or permanent nerve dysfunction occurs during the operation, the surgical strategy may be changed for safety [23]. For patients who already have unilateral vocal cord paresis, an operation on the contralateral side using IONM will help, not to prevent an RLN injury, but to detect RLN paresis, to guide postoperative care, or even to prompt the surgeon to do a protective tracheostomy after the thyroid surgery. One of our patients developed RLN paresis during the operation and we changed our strategy to a total laryngectomy with a tracheostomy.

This study has some limitations. The first is the lack of randomized control groups because we have used an IONM device for every patient given a reoperation since it was introduced. Therefore, we used as our control group patients operated on before the introduction of the IONM. It is almost impossible to do a prospective randomized control study for this topic for three reasons. First, there are not many such cases every year. Second, if we select cases from several surgeons, differences in technique will significantly bias the results. Third, it is unethical to put high-risk patients into a control group that does not allow the surgeon to use currently available technology to help identify the route of the nerve so that it will not be accidently damaged. Therefore, this study presents the usefulness of IONM during thyroid surgery done by a single high-volume surgeon. The second is that we did more reoperation after introduce of IONM since 2006 (15 cases in 5 years *vs.* 56 cases in 5 years). Before introduction of the device, we reoperated in a limited indication. And the percentage of thyroid cancer is higher in the group without IONM usage. After IONM usage, we must say that we gain more assurance and widen the indication of reoperation to recurrent benign goiter or Graves’ disease. In our experience, avoiding direct dissection over the RLNs is the best way to prevent injury. The IONM helps to identify or map the route of RLNs, but it does not always prevent the surgeon from transecting the nerve.

## Conclusion

This is the first paper that clearly states the influence of IONM on a single high-volume surgeon during re-exploration of the RLN. Using IONM for re-exploring RLNs lowers the nerve-injury rate. In addition, it may help guide surgical strategy and even help with nerve re-anastomosis. Therefore, we recommend using IONM for this high-risk group of patients.

## Abbreviations

IONM: Intra-operative nerve monitoring; RLN: Recurrent laryngeal nerve.

## Competing interests

The authors declare they have no competing interests.

## Authors’ contributions

YC participated in the design of the study and performed the statistical analysis, participated in the sequence alignment, and drafted the manuscript. SH conceived of the study, and participated in its design and coordination and helped to draft the manuscript. Both authors read and approved the final manuscript.
